# Sex, Attractiveness, and Third-Party Punishment in Fairness Consideration

**DOI:** 10.1371/journal.pone.0094004

**Published:** 2014-04-07

**Authors:** Jia Li, Xiaolin Zhou

**Affiliations:** 1 Research Center for Learning Science and Key Laboratory of Child Development and Learning Science (Ministry of Education), Southeast University, Nanjing, China; 2 Center for Brain and Cognitive Sciences and Department of Psychology, Peking University, Beijing, China; 3 Key Laboratory of Machine Perception (Ministry of Education), Peking University, Beijing, China; 4 PKU-IDG/McGovern Institute for Brain Research, Peking University, Beijing, China; University of Zaragoza, Spain

## Abstract

Social evaluation of others is often influenced by the physical attractiveness of the person being judged, leading to either a beauty premium or penalty depending on the circumstances. Here we asked Chinese participants to act as an interest-free third party in a dictator game and to evaluate the fairness level of monetary allocation by attractive and less attractive proposers of the same or opposite sex. We also instructed participants to express their willingness to punish the proposers by using a visual analogue scale. Results confirmed that the reasonableness evaluation was mainly affected by the reasonableness of offers. However, participants' intention to punish the proposers was affected by the level of reasonableness in the asset distribution and by both the sex and attractiveness of the proposers. Overall, male proposers were punished more severely than female proposers. Moreover, the same-sex proposers were punished more severely than opposite-sex proposers when they were physically attractive; this pattern was reversed when the proposers were less physically attractive. These results demonstrate social responses following an individual's unfair asset distribution can be affected by both social norms and the personal characteristics of the individual.

## Introduction

An individual's biological sex and physical appearance are the most obvious and accessible personal characteristics in social interactions [1]. Although we are taught not to “judge a book by its cover”, we are constantly influenced or biased by the physical attractiveness of the individuals we interact with. Attractive people are generally regarded as more amicable, helpful, trustworthy, intelligent, socially skilled, and hence, receive more favorable treatments than less attractive people [2], [3]. This “beauty premium” can be observed in a variety of situations, including romantic relationships [4] and job-related situations [5]. However, studies also showed that physical attractiveness might come along with a “beauty penalty”. For example, attractive people may receive less positive evaluations or treatments from people of the same sex than from people of the opposite sex. This negative bias against attractive, same-sex people has been shown to be automatic and powerful [6]–[8], and can be found both in interpersonal relationships and in organizational settings [9]–[11].

In economic exchanges, attractive people are often offered more than less attractive ones, as seen in the ultimatum game [12] and the trust game [13]. Individuals are more likely to cooperate with those they find attractive in the prisoner's dilemma and public goods games [14], [15]. However, when people find out that the certain attractive people do not contribute more than others to the greater good, they tend to invest less in these people than the less attractive people in the following round of investment [13], [14].

Participants in the above economic exchange studies played either as proposers of asset distributions or as thrusters/investors, and hence are interest-relevant parties in financial settlements. Moreover, all of the studies were carried out in the Western cultures with “positive” dependent variables (i.e., variables related to job hiring, monetary reward, or desire for social interaction). It is not clear whether the patterns of beauty premium and beauty penalty would manifest in a different culture, when the dependent variables are “negative”, and/or when the decision makers are an interest-free third party. Here we investigated to what extent Chinese participants, acting as an interest-free third party, behave differently towards attractive and less attractive individuals in economic exchanges, particularly when these individuals are unfair to others.

Previous studies find that humans tend to be highly averse to inequity in asset distribution and are willing to inflict punishment upon individuals who behave unfairly towards others [16]–[18], even when there is no previous relationship or potentially compromising interest [19], [20]. The manipulation of equity allows us to investigate whether the third-party punishment would be affected by the attractiveness of individuals who make (un-)fair offers to others in asset distribution, whether the potential beauty premium or penalty would be modulated by the congruence of sex between the proposers and the third-party participants, and to what extent these subjective attractiveness effects can be dissociated from the real perception of fairness in asset distribution.

In our experiment, participants observed a Dictator Game (DG) in which proposers (either attractive or less attractive) made offers to anonymous recipients. The participants, acting as the interest-free third-party, evaluated the reasonableness of the offers and expressed their intention (or lack thereof) to punish the proposers. Based on previous studies [10], [11], we predicted that unreasonable offers would incur greater punishment than reasonable offers (*Hypothesis 1*); more specifically, attractive people who made unfair offers would receive greater punishment than less attractive people (i.e., a beauty penalty effect), and attractive people would also receive more punishment from the same-sex participants than from the opposite-sex participants (i.e., a sex bias effect) (*Hypothesis 2*). In contrast, the expectation regarding less attractive targets would have been that the biasing effects would not emerge or would be diminished (*Hypothesis 3*). Additionally, given that fair offers are considered to be “normal” and given that there was no real reason and thus no legitimation to punish the proposers for fair offers to the recipients, it would be likely that participants' behavior in the fairness conditions should differ from that in the unfairness conditions, at least in the intensity of willingness to punish the proposer. So we predicted that the effect which emerged for the unfair offers should be mitigated or eliminated in the conditions of the fair offers (*Hypothesis 4*).

## Material and Methods

### Participants

A total of fifty-nine undergraduate and graduate students (30 females, mean age 22.88 years, *SD* = 1.54), 39 from Beijing University of Posts and Telecommunications and 20 from China Agricultural University, were tested. These two groups of participants were tested separately, with an interval of about 9 months. All the participants were healthy with no report of emotional or psychological disorders and were paid 25 Chinese Yuan (about US $4) for their participation. Informed written consent was obtained from each participant before the test. This study was carried out in accordance with the Declaration of Helsinki and was approved by the Ethics Committee of the Department of Psychology, Peking University.

### Design and materials

This experiment used a 2 (participant sex) ×2 (proposer sex) ×2 (proposer attractiveness: attractive vs. less attractive) ×2 (relative fairness level: fair vs. unfair) mixed factorial design, with participant sex as a between-participant factor and proposer sex, proposer attractiveness and fairness level of offer as three within-participant factors. The participant sex and proposer sex could also be collapsed to form a variable called “sex congruence”, with the proposers and the participants being of the same or of the opposite sex.

For proposer attractiveness, we asked 14 undergraduate students (7 females, mean age 19.46 years, *SD* = .97) who did not participate in the formal experiment, to rate the physical attractiveness of 1920 people (half females) in digital passport-style photos with no smiling or background variables on a 5-point Likert scale, ranging from “completely unattractive” (1) to “very attractive” (5). All the photos were taken from a database of undergraduate students (about 18 to 22 years old) in a third university, and thus the people in photos were unfamiliar to the raters. These photos, after being slightly adjusted for color and lightness, were randomly presented on the computer screen to the raters, who were asked to input a rating score to each photo. Each rater viewed all the photos in a unique sequence. The pretest lasted about 50 minutes and the raters were allowed to have breaks during the rating.

Based on the rating, 108 photos were selected for the formal experiment, with 27 for each combination of sex and attractiveness. The people pictured in the photographs were the alleged proposers in the Dictator Game. Each attractive photo had a mean rating score higher than 3.5 while each less attractive photo had a mean rating score less than 1.5. The differentiation of attractive and less attractive photos was confirmed by the 59 participants who participated in the formal experiment and who were asked, after the experiment, to rate the photos in post-experiment checking.

For the 27 photos in each combination of sex and attractiveness, each photo was randomly paired with a “fair” or “unfair” monetary allocation scheme, with 12 photos in total for fair schemes, 12 for unfair schemes, and 3 for filler schemes. For fair schemes, the proposer would offer 50 or 40 Yuan (6 trials each) out of 100 Yuan to the recipient (the 5/5 and 6/4 schemes, respectively) while keeping the remaining part to him/herself; for unfair schemes, the proposer would offer 10 or 20 Yuan (6 trials each) out of 100 Yuan to the recipient (the 9/1 and 8/2 schemes); for the filler trials (3 trials), the proposer would offer 30 Yuan out of 100 Yuan to the recipient. Given that the number of filler trials was rather small and given that it was difficult to classify these trials as “fair” or “unfair”, we did not include these trials in data analysis.

The 108 trials were randomly assigned to a test sequence for each participant, with the restriction that no more than 3 consecutive trials were from the same experimental condition. The experiment lasted about 30 minutes for each participant.

### Procedure

Participants were tested in groups of 10 or less, with each participant being seated in front of a computer screen in a circled space. When participants arrived at the test room, they were given oral standardized task instructions and then viewed instructions again on the computer screen. They were told that they would take part in a series of studies. We then explained the rules of Dictator Game to the participants and stressed only proposers have the right to make an allocation, hence, only the proposers have non-blurred photos (we blurred the photos of the recipients). Being a third-party, the task of participants was to evaluate the reasonableness (fairness) of proposers' allocations and to express the punishment intention to each proposer. According to feedback from participants after the experiment, the participants did not perceive the influence of attractiveness in the experiment until they were told to complete post-experiment attractiveness rating after the formal experiment.

In each trial, participants viewed a photo of the 100 Yuan banknote at the center of the screen for 500 ms and then two participant's photos, one blurred and one unblurred. Participants were informed that the proposer divided 100 Yuan between him/herself and the recipient, who had to accept the proposal. The amounts of money allocated to the proposer and the recipient were indicated with a number under each photo. This allocation scheme was presented for 2500 ms, followed by a frame which contained a line of instruction (“please rate the reasonableness of this division scheme”) and the corresponding visual analogue scale at the center of the screen. Participants used the computer mouse to move a cursor on the scale and to confirm their evaluation by clicking the mouse button. The “reasonableness” scale had a mark of “−5” at one end, “+5” at the other end and “0” in the middle. Another frame with the instruction (“to what extent do you want to punish the proposer”) after which the participant interacted with the visual analogue scale to make a decision. The “punishment intention” scale had a mark of “0” at one end, “10” at the other end and “5”in the middle. The two subjective ratings had no time limit although the participants regularly completed each task within 1000 ms.

## Results

Post-experiment rating revealed that the scores for the attractive people (*M* = 3.73; *SD* = .28) and for the less attractive people (*M* = 1.62; *SD* = .31) differed significantly (*t* (58) = 40.21, *p*<.001), consistent with the result of pre-experiment rating.

As indicated earlier, the experiment was run twice, once with 39 participants and another time with 20 participants. Given that the two runs produced the same pattern of effects, we reported the results of the combined data analysis in the following paragraphs.

### Reasonableness rating

The results of reasonableness rating clearly confirmed that our manipulation of equity succesfully induced the participants' sense of reasonableness, since participants rated fair offers as far more reasonable than unfair offers. Analysis of variance (ANOVA) was performed on the rating scores (Table 1), with proposer sex, proposer attractiveness, and reasonableness of offer as three within-participant factors and participant sex as a between-participant factor. Only the main effect of fairness reached significance, *F* (1, 57) = 4829.84, *p*<.001, η_p_^2^ = .988, with fair offers being rated as more reasonable (*M* = 4.04, *SD* = .65) than unfair offers (*M* = -3.87, *SD* = .81). The main effect of proposer attractiveness and proposer sex were only marginally significant, *F* (1, 57) = 3.97, *p* = .051, η_p_^2^ = .065, and *F* (1, 57) = 3.98, *p* = .051, η_p_^2^ = .065, respectively.

**Table 1:** Mean scores for participants' evaluation of the fairness of offers made by attractive and less attractive proposers of the same or the opposite sex (Standard deviations are in parentheses).

	Fair offers				Unfair offers			
Participant	Attractive proposers		Less attractive proposers		Attractive proposers		Less attractive proposers	
	Male	Female	Male	Female	Male	Female	Male	Female
Male	4.01	4.12	3.96	4.02	−4.11	−3.82	−4.32	−3.92
	(0.85)	(0.82)	(0.76)	(0.90)	(0.52)	(0.70)	(0.43)	(0.83)
Female	4.05	4.16	4.00	4.01	−3.77	−3.51	−3.63	−3.88
	(0.39)	(0.50)	(0.49)	(0.46)	(0.78)	(0.88)	(1.67)	(0.66)

### Punishment intention rating

ANOVA with the same factors above revealed a significant main effect of fairness level of offer, *F*(1, 57) = 1249.95, *p*<.001, η_p_^2^ = .956, and a significant main effect of proposer sex, *F*(1, 57) = 32.62, *p*<.001, η_p_^2^ = .364. Participants were generally more willing to punish proposers who made unfair offers (*M* = 6.47, *SD* = 1.19) than proposers who made fair offers (*M* = 1.39, *SD* = .65), and more willing to punish male (*M* = 4.20, *SD* = .90) than female proposers (*M* = 3.67, *SD* = .93). The main effect of attractiveness was not significant, F (1, 57) = 1.48, *p*>.1, η_p_^2^ = .025, but its interactions with other factors were significant.

Importantly, fairness level of offer interacted with proposer sex, *F*(1, 57) = 23.93, *p*<.001, η_p_^2^ = .296, with proposer sex and participant sex, *F*(1, 57) = 44.90, *p*<.001, η_p_^2^ = .441, and with proposer sex, participant sex, and attractiveness, *F*(1, 57) = 228.93, *p*<.001, η_p_^2^ = .801. Interactions also includes proposer sex by participant sex, *F*(1, 57) = 41.13, *p*<.001, η_p_^2^ = .419; attractiveness by participant sex, *F*(1, 57) = 4.45, *p*<.05, η_p_^2^ = .072; and attractiveness sex by proposer sex by participant sex, *F*(1, 57) = 321.97, *p*<.001, η_p_^2^ = .850. These interactions indicated that the effect of attractiveness on third-party punishment (i.e., beauty penalty) was modulated by both the participant sex and the proposer sex.

**Figure 1:**
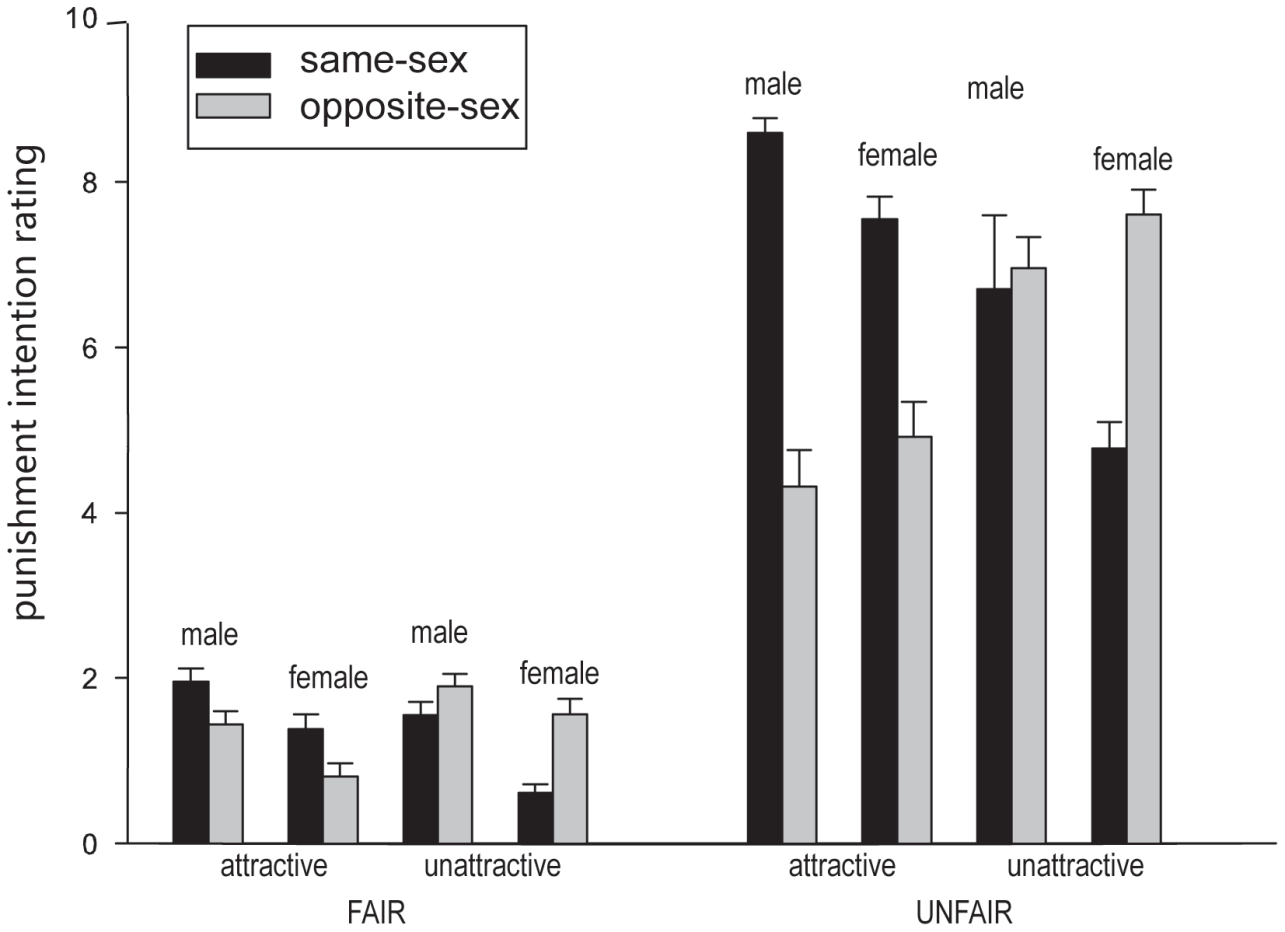
Mean scores for participants' intention to punish proposers as a function of the fairness (reasonableness) of offer, proposers' physical attractiveness and their biological sex. Same-sex  =  the proposer and the participant were of the same sex; opposite-sex  =  the proposer and the participant were of different sexes; Male  =  male participants; Female  =  female participants; Fair  =  the recipient received 50 or 40 yuan while the proposer received 50 or 60 yuan; Unfair  =  the recipient received 10 or 20 yuan while the proposer received 90 or 80 yuan. Error bars represent standard errors.

To clarify how proposers' facial attractiveness and the proposers' and the participants' sex modulated the participants' intention to punish unfair (and fair) offers, separate analyses were conducted for the punishment intention rating in the fair and unfair offer conditions. For unfair offers (Figure 1; right part), there was a significant main effect of proposer sex, *F*(1, 57) = 32.67, *p*<.001, η_p_^2^ = .364, indicating that male proposers received higher punishment scores (*M* = 6.94, *SD* = 1.40) than female proposers (*M* = 6.00, *SD* = 1.53), consistent with the overall pattern for the proposers. The interaction between proposer sex and participant sex was significant, *F*(1, 57) = 49.58, *p*<.001, η_p_^2^ = .465. Specifically, male participants, on average, exerted higher levels of punishment on male proposers (*M* = 7.66, *SD* = .92) than on female proposers (*M* = 5.57, *SD* = 1.76), but female participants exerted generally equal punishment on male (*M* = 6.22, *SD* = 1.43) and female proposers (*M* = 6.44, *SD* = 1.14). The main effect of attractiveness was not significant, *F*(1, 57) = 1.54, *p*>.1, η_p_^2^ = .026, neither was the interaction between attractiveness and proposer sex, *F*(1, 57) = .03, *p*>.1, η_p_^2^<.000. However, the three-way interaction between attractiveness, proposer sex, and participant sex was significant, *F*(1, 57) = 354.54, *p*<.001, η_p_^2^ = .861. Male participants indicated a higher intention to punish attractive male proposers (in comparison to female proposers), whereas female participants indicated a higher intention to punish attractive female proposers (in comparison to male proposers).

Regarding less attractive proposers, male participants did not show a significant difference in their intention to punish male and female proposers, while female participants exerted *lower* punishment on female than on male proposers. This observation was substantiated by further statistical tests.

For the fair offers (Figure 1; left part), we found a three-way interaction between attractiveness, proposer sex, and participant sex, *F*(1, 57) = 38.36, *p*<.001, η_p_^2^ = .402. It is clear from the left part of Figure 1 that male participants had stronger intention to punish attractive male proposers than attractive female proposers and female participants had stronger intention to punish less attractive male proposers than less attractive female proposers. This pattern of sex congruence effect was in general consistent with the pattern observed when the proposer made unfair offers.

**Figure 2:**
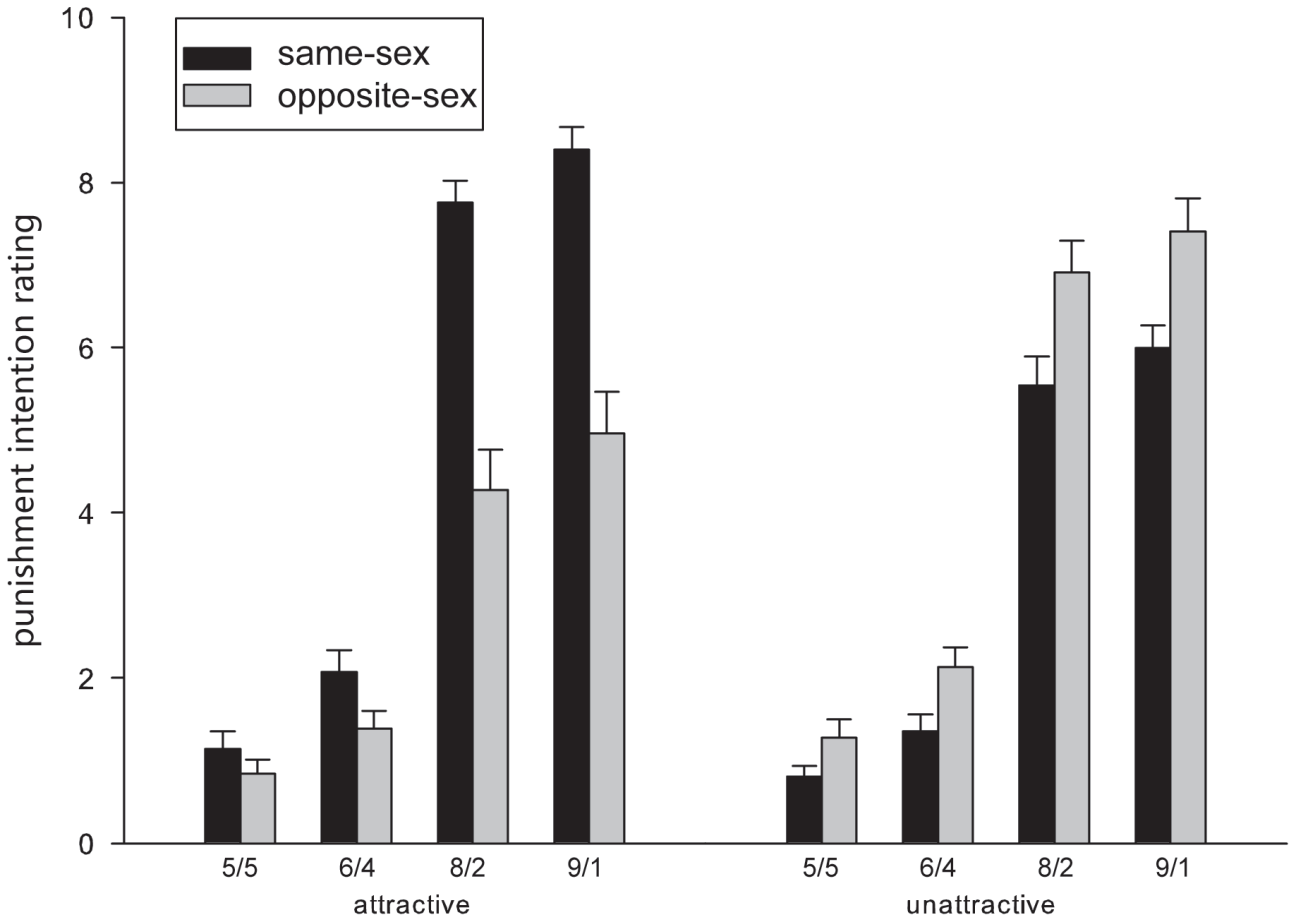
Mean scores for participants' intention to punish attractive and less attractive proposers as a function of the reasonableness of offer and sex congruence between the proposers and the participants. 5/5  =  equal division of 100 yuan between the proposer and the recipient; 6/4  =  the proposer received 60 yuan while the recipient received 40 yuan; 8/2  =  the proposer received 80 yuan while the recipient received 20 yuan; and 9/1  =  the proposer received 90 yuan while the recipient received 10 yuan. Error bars represent standard errors.

It was surprising that proposers were punished even when they made fair offers to recipients. To make sure that this punishment was not due to the inclusion of the 6/4 distribution scheme as being “fair” and to examine in more detail the sex congruence effect, we recomputed the rating of punishment intention as a function of more detailed categorization of division schemes (i.e., categorizing the fair offers further into the 5/5, 6/4 levels and the unfair offers into the 8/2, 9/1 levels); however, this time we grouped the scores according to whether the proposers and the participants were of the same sex (male-male, female-female) or of opposite sex (male-female, female-male). Figure 2 illustrates the mean scores in different conditions.

ANOVA with level of fairness (5/5 vs. 6/4 vs. 8/2 vs. 9/1), level of attractiveness (attractive vs. less attractive), sex congruence between the proposers and the participants (same vs. different) as three within-participant factors revealed a significant main effect of fairness level, *F*(3, 174) = 711.80, *p*<.001, η_p_^2^ = .925. The differences between the reasonableness ratings for the four levels (mean scores being 0.93, 1.87, 6.09 and 6.83, respectively) were all significant, *ps*<.001. The main effect of sex congruence was also significant, *F*(1, 58) = 32.57, *p*<.001, η_p_^2^ = .360, with the punishment intention being stronger when the participants and the proposers were of the same sex (*M* = 4.24, *SD* = .88) than of a different sex (*M* = 3.62, *SD* = .97). Importantly, this congruence effect was modulated by the proposers' physical attractiveness, by the fairness of the offers, and by attractiveness and fairness jointly, as the two-way and the three-way interaction were all significant, *F*(1, 58) = 300.30, *p*<.001, η_p_^2^ = .838; *F*(3, 174) = 19.70, *p*<.001, η_p_^2^ = .254, and *F*(3, 174) = 71.88, *p*<.001, η_p_^2^ = .553, respectively.

Further analyses were conducted for the intention scores of punishing attractive and less attractive proposers, respectively. For attractive proposers, the main effect of sex congruence was significant, *F*(1, 58) = 175.22, *p*<.001, η_p_^2^ = .751, with a stronger punishment intention for the same-sex participants (*M* = 4.85, *SD* = .85) than for the opposite-sex participants (*M* = 2.93, *SD* = 1.09). Moreover, the interaction between sex congruence and fairness level was significant, *F*(3, 174) = 76.35, *p*<.001, η_p_^2^ = .568, indicating that the sex congruence effect was smaller for fairer offers (0.31 for the 5/5 scheme, 0.55 for the 6/4 scheme) than for unfair offers (3.46 for the 8/2 scheme, 3.36 for the 9/1 scheme). For less attractive proposers, the main effect of sex congruence was also significant, *F*(1, 58) = 31.83, *p*<.001, η_p_^2^ = .354, with the punishment intention being *weaker* for the same-sex participants (*M* = 3.63, *SD* = 1.01) than for the opposite-sex participants (*M* = 4.30, *SD* = 1.00). The interaction between sex congruence and level of fairness reached significance, *F*(3, 174) = 5.54, *p*<.01, η_p_^2^ = .087, indicating that the reversed sex congruence effect was smaller for fair offers (0.29 for both the 5/5 and the 6/4 schemes) than for unfair offers (1.02 for the 8/2 scheme, and 1.09 for the 9/1scheme).

## Discussion

By presenting photos of attractive or less attractive proposers who made fair or unfair offers to recipients in a dictator game to participants acting as a third-party punisher, we found that Chinese participants in general had harsher feelings towards individuals who made unfair offers than towards those who made fair offers (*Hypothesis 1* confirmed); however, the strength of this intention to punish the proposers was modulated by the sex and attractiveness of the proposer. Overall, participants were more willing to punish male proposers than female proposers. Moreover, participant were more willing to punish attractive proposers of the same sex than attractive proposers of the opposite sex (*Hypothesis 2* confirmed) but were less willing to punish less attractive proposers of the same sex than less attractive proposers of the opposite sex (*Hypothesis 3* not confirmed). This was true for both proposers who made unfair offers and those who made fair offers (*Hypothesis 4* not confirmed).

The absence of a beauty premium or penalty effect in the evaluation of the reasonableness of offers, as oppose to the presence of this effect in participants' willingness to punish the proposers, demonstrates that our participants could make relatively objective judgments on attributes related to attractive individuals when act as an interest-free third party. This finding appears to be inconsistent with findings from earlier studies in which attractive people were generally believed to be more amicable, more socially skilled, and more trustworthy than less attractive individuals [2], [3]. However, we believe that the difference between the studies is due to the availability of critical information. When there is no objective information concerning the individuals in question, their physical attractiveness may affect other's perception and judgments of their internal attributes, such as personality, ability, and motivation/intention. By providing the participant with objective information concerning the individuals' overt (moral) behavior and the outcome related to the behavior, the participant's were objective in their perception of fairness, and this objectivity was unaffected by the individuals' personal characteristics, such as sex and physical attractiveness. Thus, it seems that social norms of egalitarian asset distribution prevail over other potential modulating factors when (moral) judgment is in concern.

Participants became more subjective when it came time for them to make a decision. Participant punishment ratings were susceptible to the influence of the proposers' sex and physical attractiveness. For attractive proposers, participants had a stronger intention to punish the same-sex proposers, as compared with the opposite-sex proposers. This finding based one a “negative” dependent variable, replicates a negative bias towards attractive same-sex people found in the Western cultures population samples [6]–[8]. Apparently, participants had a soft spot for the opposite-sex attractive people but had harsher feelings towards the same-sex attractive people, consistent with the earlier finding of automatic favorable responses towards attractive opposite-sex people (i.e., the category of potential mates [8]) and less favorable responses towards attractive same-sex people (i.e., the category of potential rivals [7]). It is possible that the participants simply react in a more positive way to attractive opposite-sex (in comparison to same-sex) people, because social comparison threat and potential rivalry are less strong and/or less likely in opposite-sex constellations (in comparison to same-sex constellations).

For less attractive proposers, however, participants were less tolerant of the opposite-sex people than of the same-sex people, and this was particularly the case for female participants. Regarding people who are not attractive, same-sex people might at least have the benefit of similarity (i.e., people tend to feel more comfortable being around same-sex individuals who are not threatening), whereas opposite-sex individuals who are not attractive elicit no positive reward for the perceiver (i.e., in terms of evolutionary psychology, they would be neither interesting as a potential mate nor would they be likely to become a friend, as most friendships are among people of the same gender). Given past research in evolutionary and social psychology showing that females are choosier regarding opposite-sex cohorts, it seems comprehensible that the female participants in the ultimatum game reacted comparably negative toward male proposers who were neither particularly good-looking nor appeared to be fair and generous.

It is possible that participants' positive and negative reactions to the proposers reflect a (potentially unconscious) desire to interact with a person or to get to know a person [11], [21]-[23]. Being unfair is a violation of social norms; if this violation is coupled with violation of the implicit expectation, this person could be negatively evaluated and receive feelings of resentment or punishment from others. Obviously, this speculation needs to be tested in further studies. But as it stands, the present study demonstrates that expectations based on social norms and expectations based on physical attractiveness or other tangible properties may interact in interpersonal relationships.

Two other findings in this study are worth discussion. The first is that participants were more willing to punish male proposers than female proposers, and this was particularly so for male participants. The second is that participants expressed their intention to punish proposers even when these proposers offered equal distribution of assets between themselves and the recipients. We suggest that these “irrational” responses may reflect people's understanding of “fairness” in a specific cultural setting. In general, males are in a more dominant position than females and, as such, they are expected to be more generous in asset distribution than females. But if males violate this expectancy or “social norm” and behave unfairly towards others, they would receive stronger punishments than females. Conversely, females tend to be perceived as “weaker”, and people, particularly males, might have a stronger inhibition threshold in punishing them, even if they have acted unfairly (in comparison to males acting the same way). Similarly, proposers are in a stronger position than recipients in asset distribution. In the Chinese culture, people in such positions are expected to be generous and altruistic. Violation of this expectancy, even as mild as equal distribution of assets between the proposers and the recipients, could incur punishment. Further studies are also needed to verify these suggestions and to examine whether these findings can be replicated in non-Chinese or non-Asian cultures.

To conclude, by asking participants acting as an interest-free third-party in a dictator game to evaluate the reasonableness of asset division proposed by attractive or less attractive people of the same or the opposite sex, we found that individuals were objective in their evaluation of reasonableness; however, when it came time to enforce punishment on the proposers, participants were affected by the sex and physical attractiveness of the proposers. Overall, male proposers were more likely to be punished than female proposers. Attractive people were more likely to be punished if they were of the same sex, whereas less attractive people were more likely to be punished if they were of the opposite sex, suggesting that social responses and negative reactions following an individual's unfair asset distribution can be affected by both social norms and the personal characteristics of that individual.
